# Indirect Effect of Pesticides on Insects and Other Arthropods

**DOI:** 10.3390/toxics9080177

**Published:** 2021-07-30

**Authors:** Francisco Sánchez-Bayo

**Affiliations:** School of Life and Environmental Sciences, The University of Sydney, Eveleigh, NSW 2015, Australia; francisco.sanchez-bayo@sydney.edu.au

**Keywords:** insecticides, herbicides, fungicides, parasiticides, pests, aquatic insects, predation, competition, ecological interactions

## Abstract

Pesticides released to the environment can indirectly affect target and non-target species in ways that are often contrary to their intended use. Such indirect effects are mediated through direct impacts on other species or the physical environment and depend on ecological mechanisms and species interactions. Typical mechanisms are the release of herbivores from predation and release from competition among species with similar niches. Application of insecticides to agriculture often results in subsequent pest outbreaks due to the elimination of natural enemies. The loss of floristic diversity and food resources that result from herbicide applications can reduce populations of pollinators and natural enemies of crop pests. In aquatic ecosystems, insecticides and fungicides often induce algae blooms as the chemicals reduce grazing by zooplankton and benthic herbivores. Increases in periphyton biomass typically result in the replacement of arthropods with more tolerant species such as snails, worms and tadpoles. Fungicides and systemic insecticides also reduce nutrient recycling by impairing the ability of detritivorous arthropods. Residues of herbicides can reduce the biomass of macrophytes in ponds and wetlands, indirectly affecting the protection and breeding of predatory insects in that environment. The direct impacts of pesticides in the environment are therefore either amplified or compensated by their indirect effects.

## 1. Introduction

Indirect effects of toxicants on organisms are defined as those mediated through direct impacts on other species or the physical environment. They derive from the ecological structure of ecosystems, where species survival depends on complex interactions that typically involve competition for resources and trophic relationships. Indirect effects can be thought of as side-effects that are related to the ecological traits of the species affected rather than to the toxic mode of action of the individual chemicals. Consequently, different classes of toxicants can have the same indirect effects on individual species and ecosystems.

Pesticides are toxic chemicals with specific modes of action, which are designed to kill organisms. The enormous variety of pesticides in the market, with thousands of chemicals in use [1], reflects the diversity of target organisms and their particular biochemical and physiological characteristics. It is well known that the application of pesticides in agriculture to control pests, weeds and fungal diseases impacts also on non-target species of plants and animals which are equally susceptible to the toxic chemicals [2,3,4,5]. Studies of these unintended impacts on arthropod communities have focused mainly on the direct effects that pesticides have on terrestrial and aquatic species. Many recent studies have also investigated the sub-lethal effects of pesticides on insects, prompted by observations of negative impacts of low residues of these chemicals on pollinators [6]. These studies are well covered in several books and reviews on the topic [7,8,9,10]. Indirect effects are sometimes reported in such studies, but most of what we know about indirect effects comes from controlled experimental mesocosm and microcosms that simulate the natural assemblages of plants and animals [11,12].

Pesticides typically reduce organisms abundance by directly increasing the mortality or reducing the fecundity of the target species [13]. In contrast, indirect effects can increase the populations of some species while reducing those of others, as these effects reflect ecological impacts caused by habitat modification, resource competition between species or cascades through the food webs [14]. If the effects persist, the final outcome is a different community structure that may result in either impaired or enhanced functionality of the ecosystem—usually the former.

Standard laboratory toxicity tests cannot detect indirect pesticide effects. Model ecosystems (e.g., microcosms and mesocosms) are needed to observe the impacts at the community or ecosystem level [15]. Just as the effectiveness of an insecticide in controlling a pest population should not be measured in terms of adult mortality alone (i.e., LC50 values), but should rather be evaluated by the negative population growth rate on the target species [16], pesticide impacts on non-target species cannot be assessed by their lethal effects alone but must consider other factors that contribute to the population declines. This is because population size is determined not only by the abundance of adults but also by their fertility rate and, in the case of arthropods, by the number of life cycles that a species has in a given year (voltinism). In addition, interactions among species, due to competition or predation, can define population-level effects of pesticides in a more complex way than is commonly thought, as increasing competition usually leads to more sensitivity to the chemicals [17]. This consideration matters, as the indirect effects of pesticides involve changes in population size among different species, not toxic effects. Experimental studies in aquatic mesocosms have shown that changing the number of species within trophic levels (horizontal composition) can either increase or decrease the effects of a given pesticide, depending on the individual species interactions. However, changing the vertical composition by adding a number of trophic levels always increased these effects [18]. In reality, the overall ecological effects of pesticides cannot be determined by the sensitivity of a single species to the toxicants, as interactions among species can reduce or amplify whatever toxic effects the chemicals may have.

Understanding the indirect impacts of pesticides is important from a managerial point of view, especially for the successful implementation of integrated pest management (IPM) tools in agriculture and forestry. About 26% of insect species are associated with the approximately 310,000 species of vascular plants [19], while more than 70 families of arthropods known to be potential crop pests are primarily associated with weeds [20]. Weber et al. [21] suggest that weed scientists and entomologists need to work together to more thoroughly understand the effect of weed management on insect population dynamics — unfortunately, this rarely happens, to the detriment of IPM practices. Since the goal of pest management is to control pest populations to levels that don’t cause economic harm, it is essential to know what factors intervene in the outbreak of individual pest species. Apart from unusual seasonal weather patterns that can trigger an explosive population growth in some arthropods, the disruption of natural controls systems is known to be a crucial factor in pest outbreaks. Pesticides can cause such disruption by reducing drastically the populations of predators, parasites and pathogenic organisms which control the incipient pests [22,23,24]. Restoring the ecosystem balance is not always possible, as prolonged effects of persistent chemicals may deter the re-establishment of the original community structure [25,26]. It is for this reason that a sound knowledge of the ecological processes involved in pesticide impacts, including their direct and indirect effects on species and communities, matters.

This article aims at putting together what we know about the indirect effects that different types of pesticides have on insects and other arthropods. This review is not intended to be exhaustive, as much of what is described here has already been reported in previous reviews on pesticides impacts [13,27,28]. The focus here is on the ecological mechanisms underpinning such effects, which may explain the various outcomes obtained in each case.

Because arthropod communities differ markedly between terrestrial and aquatic ecosystems, the exposition below refers separately to the impacts on these two contrasting environments.

## 2. Indirect Effects in Terrestrial Ecosystems

**Figure 1:**
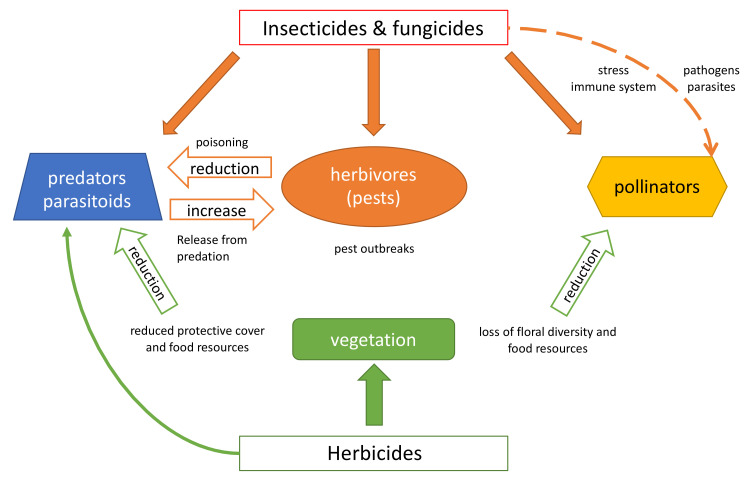
Indirect effects of pesticides on terrestrial arthropods are indicated by hollow and dashed arrows and direct effects by solid arrows.

Pesticides are mainly applied to terrestrial ecosystems, as their primary objective is to control animal pests, weeds and diseases in agricultural production. We start our discussion therefore by considering the indirect effects of various classes of pesticides on terrestrial environments (Figure 1). Note that rodenticides are not included here because they do not affect arthropods, but only vertebrate species.

### 2.1. Herbicides

Impacts of herbicides on insects and other arthropods often result from the elimination of the target and non-target host plants. Although cultivation has also negative effects on many soil organisms for the same reason, conservation tillage to avoid soil erosion has increased herbicide usage in many countries [29]. The increase is concerning, as the half-life of herbicides in the environment often exceeds a month and for some compounds is more than 1 year, so insects within fields and adjacent borders exposed to drift are most likely to be exposed to herbicides in soil [28]. Most reports on the impact of herbicides indicate alterations in insect survival or egg production due to an increase or decrease in host plant population as an indirect effect [30]. The reduction in predatory arthropods [31] and pollinators [32,33] are in many cases due to the removal of plant hosts, pollen and nectar, shelter, nesting and overwintering sites by the herbicides [34,35].

In contrast to insecticides or fungicides, some herbicides may be compatible with biological agents to control weeds, for example, 2,4-D when combined with the weevil *Trichosirocalus horridus* (Coleoptera: Curculionidae) to control thistles [36]. This is because the lethal doses for the insects are much higher than those required to control the target weeds. Indeed, most herbicides are not acutely toxic to soil organisms, except perhaps some triazine and dinitroaniline herbicides and TCA-sodium and monuron at very high doses. For example, 10% of dichlobenil (DCBN or 2,6-dichlorobenzonitrile) has negative effects on the reproductive rate, biomass and abundance of the aphid *Sitobion avenae* (Hemiptera: Aphididae) [37], and some 54% of herbicides are harmful or moderately harmful to parasitic wasps and other beneficial arthropods [38]. In addition, pronamide, propham and simazine have antifeedant properties on various phytophagous pests [27], and various oils used as adjuvants or carriers for 2,4-D are themselves toxic to honeybees [39]. 

Early evidence of indirect effects on insect populations came from the UK. An investigation on the decline in grey partridges found that chicks mortality increased due mostly to lack of insects in herbicide treated fields, although the direct elimination of insects by insecticides was an additional factor [40,41]. In Europe, there is evidence that yearly application of herbicides to cereal fields since 1970 has resulted in losses of many arthropod and weed species [42], and declines in carabid populations were related to a number of factors including the increase in the area sprayed with herbicides and insecticides [43]. Effects on the abundance of soil invertebrates vary among herbicides and taxa. Atrazine decreased the abundance of wireworms and springtails whereas dalapon and TCA appeared to increase millipedes, springtails and mites, and 2,4-D had little or no effect, mainly due to loss of floristic diversity and food availability [44,45]. The herbicides 2,4-D and glyphosate applied in combination to non-tillage corn fields reduced significantly the abundance of spiders, mites, crickets and ground beetles, whereas conventional cultivation practices did not [46].

Increases in pest species due to a reduction in natural enemies by herbicide treatments have been documented. Some reductions are due to negative side effects of the chemicals on predatory and parasitoid species. For example, the overall parasitism rate by specialist wasps, *Aphidius rhopalosiphi* (Hymenoptera: Braconidae) decreased in plants treated with 0.1% of DCBN, with the herbicide introducing also a sex ratio bias [37]. Although most herbicides probably have a very little direct effect on arthropod populations, treatments for weed control with 2,4-D indirectly increased the density of sugarcane borer pests, and this was attributed to reductions in the parasitoid *Trichogramma minutum* (Hymenoptera: Trichogrammatidae) caused by direct toxicity of the herbicide [47]. Predatory mites are more sensitive to several herbicides, including paraquat, 2,4-D, terbacil, and dalapon, than are the phytophagous mites, so the application of these herbicides favours the pest species over their predators [27]. Application of toxaphene herbicide reduced beneficial insects and increased phytophagous insects in comparison with non-treated control plots [48]. In other cases, the reasons for the pest outbreaks are not clear. For example, contrary to the intended purpose, solutions of paraquat at 1 or 3% applied to plantations of slash pine (*Pinus elliotti*) increased the attack by black turpentine beetle, *Dendroctonus terebrans* (Coleoptera: Curculionidae) and engraver beetle pests, *Ips* sp. (Coleoptera: Curculionidae) on the treated trees [49]. In another case, the damage of the wireworm *Ctenicera aeripennis destructor* (Coleoptera: Elateridae) to wheat was increased when 2,4-D was applied prior to sowing the crop, probably because the crop lost vigour and was less able to withstand the wireworm damage [50]. Similarly, aphid and corn borer populations increased in corn crops following the application of 2,4-D [51].

More commonly, the increase in pest populations results from the elimination of floristic diversity and alteration of the plant community structure, which harbours parasitic and predatory arthropods [52]. This has been reported for ground predators such as *Loricera pilicornis* and *Agonum dorsale* (Coleoptera: Carabidae) [53], with some authors suggesting that the decline of carabid populations is probably due to reduction in food or cover rather than the direct effect of herbicides [54]. However, a large study on 100 cereal fields in the UK found carabid populations fluctuated between years but showed no consistent trend [55]. Carabid beetles have been extensively studied, primarily because of their role as pest predators and also because they are easily sampled within pesticide field trials. Indeed, altering the floral composition and structure of field boundaries changes their suitability as nutritional sources or overwintering sites for many insects. Insect larvae can also be more numerous in weedy areas because conditions for egg-laying or larval survival may be more favourable as a consequence of the increased humidity and protection from predators provided by the vegetation [43,56]. Both males and females of the meadow grasshopper, *Pseudochorthippus parallelus* (Orthoptera: Acrididae) reduced herbivory but increased the number of offspring in laboratory cages with several grass mixtures and herbicides, suggesting that herbicides may shift resource allocation among generalist insect herbivores [57].

Reductions in floristic diversity by herbicides have an indirect negative impact on butterflies and bee pollinators by limiting the amount of available resources [58]. Declines of butterflies have been linked to herbicide use in the UK, as transect surveys of butterflies in cereal fields treated with conventional applications of herbicides, fungicides and insecticides rendered significantly lower abundance of 13 butterfly species than in field margins where pesticides were not applied: a total of 868 butterflies were recorded on the unsprayed plot compared to 297 on the sprayed plot [59]. In America, a 58% decline in milkweeds on the Midwest landscape and an 81% decline in populations of the monarch butterfly, *Danaus plexippus* (Lepidoptera: Nymphalidae) from 1999 to 2010 have been documented. This loss is coincident with the increased use of glyphosate herbicide in conjunction with increased planting of genetically modified (GM) glyphosate-tolerant corn and soybeans [60]. Similarly, dicamba reduced the biomass of thistles, and consequently, caterpillars of *Vanessa cardui* (Lepidoptera: Nymphalidae) that fed on young plants grew smaller as well, possibly due to a lack of essential nutrients. However, no effects were observed on adult butterflies [61]. Experimental trials show that plants exposed to sublethal doses of glyphosate flowered at shorter heights and produced shorter leaves, while the size of the floral display had the largest effect on insect visitation, with larger floral displays significantly more likely to receive a visitor in a given sampling event [62].

### 2.2. Insecticides

Four main indirect effects have been reported in the literature since the widespread use of insecticides begun in the 1940s. Two of them concern the pest control operations, which often went awry for not taking into account the underlying ecology of the agroecosystems. Another is linked to poisoning through the food chain, and a final indirect effect is related to the stress that toxic chemicals induce on organisms.

#### 2.2.1. Pest Resurgence

Since insecticides are used to control pest populations of insects and other arthropods, pest resurgence is totally contradictory to the intended outcome of their application. And yet, pest resurgence is the best documented indirect effect of insecticides. Hardin et al. [63] stated that “insecticide-induced resurgence of arthropod pests has long been known to occur in response to a reduction in natural enemy populations, releasing the pest population from regulation.” The reduction or elimination of the natural predators and parasitoids by the insecticides applied are the primary cause of the pest resurgence. Other mechanisms, such as enhancement of pest fecundity by more productive crop varieties, altered host-plant nutrition, or increased attractiveness may also contribute to resurgence [63], but are not the main cause.

When DDT was first applied in California against the citrus red scale, *Aonidiella aurantii* (Hemiptera: Diaspididae), the insecticide also eliminated the wasp parasitoid *Aphytis melinus* (Hymenoptera: Aphelinidae) that was the natural agent to control that pest. Populations of the scale doubled within a year and exploded out of control in the following years [64]. Predatory ants *Ectatomma ruidum* (Hymenoptera, Formicidae) can efficiently remove pupae of the fall armyworm, *Spodoptera frugiperda* (Lepidoptera: Noctuidae), a main pest of corn. However, when corn crops in Nicaragua were treated with carbofuran, the population of this pest increased because the ants foraging activity was significantly curtailed due to the insecticide [65]. In a classic case of pest resurgence, cypermethrin applied to wheat fields in England increased populations of thrips and collembola due to the suppression of their predators, in particular carabid beetles and *Tachyporus* sp. larvae (Coleoptera: Staphylinidae) [66].

#### 2.2.2. Outbreak of Secondary Pests

Secondary pest outbreaks are the indirect effect of eliminating the primary pests of crops, and therefore result from a release from competition among herbivore species. Outbreaks of secondary pests in cotton are well known since the introduction of DDT in the late 1950s in Sudan, Mexico and later in Australia. The main cause of such outbreaks was the suppression of secondary pests’ natural enemies, including predators and parasitoid wasps that kept at bay the populations of secondary pests such as the silverleaf whitefly, *Bemisia tabaci* (Hemiptera: Aleyrodidae) [67,68].

Susceptibility of the pest species to insecticides is key to understand this replacement. In experimental studies, the dominance of the maize weevil, *Sitophilus zeamais* (Coleoptera: Curculionidae) in cereal crops shifted to the lesser grain borer, *Rhyzopertha dominica* (Coleoptera: Bostrichidae) under exposure to fenitrothion, indicating that the insecticide indirectly mediated the species interactions and shifted their population densities, raising concerns as a potential cause of secondary pest outbreaks [69]. When chlorpyrifos was applied to corn crops for the control of *S. frugiperda*, it also significantly reduced the foraging activity of predatory ants. The suppression of both the main pest and its primary natural control agent resulted in higher levels of the maize leafhopper, *Dalbulus maidis* (Hemiptera: Cicadellidae), a secondary pest of that crop [65]. In another example, the application of chlorpyrifos to vineyards led to higher densities of spider mites, *Tetranychus* sp. (Acari: Tetranychidae), because the predatory phytoseiid mites were eliminated by the insecticide [70].

The replacement of commercial crop varieties of cotton and corn with GM Bt-cotton and Bt-corn (Bt-crop refers to the genetically modified crops that express the delta-endotoxins found in *Bacillus thuringiensis*) meant the removal of the main pests of those crops, such as the cotton bollworm, *Helicoverpa* sp., (Lepidoptera: Noctuidae), the armyworm, *Spodoptera* sp. (Lepidoptera: Noctuidae), corn rootworm, *Diabrotica* sp. (Coleoptera: Chrysomelidae), corn borers, *Ostrinia* sp. (Lepidoptera: Crambidae) and maize weevil, *S. zeamais*. Widespread adoption of Bt cotton, while having reduced the number of insecticide sprays on cotton fields [71], has resulted in an increased abundance of stink bug pests such as *Euschistus servus* and *Nezara viridula* (Hemiptera: Pentatomidae), which cause considerable damage as well [72]. Indeed, the GM varieties have allowed other herbivorous insects to flourish and become pests in those crops, i.e., aphids, whiteflies, stink bugs, etc. At the same time, the population size and species richness of parasitoids might decrease due to the lower density of their formerly abundant host pests [73]. Similarly, the use of insecticides in rice crops over many years has led to the suppression of the main rice pests, such as stemborers, *Scirpophaga* sp. and *Chilo* sp. (Lepidoptera: Crambidae) and the rice leafroller, *Cnaphalocrocis medinalis* (Lepidoptera: Crambidae), and their replacement with brown planthoppers, *Nilaparvata lugens* (Hemiptera: Delphacidae) and several rice bugs, *Leptocorisa* sp. (Hemiptera: Alydidae), which are the dominant pest nowadays [74]. Secondary pests are also costly, with early-season applications of broad-spectrum insecticides to control the plant bug, *Lygus hesperus* (Hemiptera: Miridae) a secondary pest in cotton, costing an additional 20% overall costs to farmers in the San Joaquin Valley of California [75].

#### 2.2.3. Secondary Poisoning of Natural Enemies

Target pests and other non-target insects are usually decimated when insecticides are applied to a crop, but individual insects don’t die instantly: the time to death can vary from a few minutes to a few days depending on the exposure dose each insect receives. In the meantime, natural enemies feeding on the affected species may experience secondary poisoning and alter their predatory ability or even die. For example, predation of the spined soldier bug, *Podisus maculiventris* (Hemiptera: Pentatomidae) was impaired and its weight gain lowered as the bugs fed on diamond moths, *Plutella xylostella* (Lepidoptera: Plutellidae) in cabbage plots treated with imidacloprid; interestingly, the insecticide did not significantly reduce the moth numbers despite having been applied at the recommended rates [76].

Secondary poisoning more commonly results in mortality of the natural enemies in both laboratory and field experiments. Thus, survival of larvae of the lacewing *Mallada signatus* (Neuroptera: Chrysopidae) was reduced in laboratory trials after feeding on second-instar larvae of cotton bollworms, *Helicoverpa armigera* (Lepidoptera: Noctuidae) that had been treated with azadirachtin [77]. Equally, large proportions of larvae of the lacewing *Micromus tasmaniae* (Neuroptera: Hemerobiidae) died after feeding on lettuce aphids, *Nasonovia ribisnigri* (Hemiptera: Aphididae) treated with various systemic insecticides in the laboratory, with imidacloprid treatments causing 96% mortality and pirimicarb 30–40% mortality [78]. Survival of the ladybug *Cycloneda sanguinea* (Coleoptera: Coccinellidae) fed on aphids treated with thiamethoxam and imidacloprid was also significantly reduced in both laboratory and field settings [79], while residues of dimethoate in prey aphids that had been treated at field exposure rates caused significant mortality levels in three carabid predators: *Pterostichus madidus, P. melanarius* and *Nebria brevicollis* (Coleoptera: Carabidae) [80]. This indicates that carabids feeding in treated fields and field margins could suffer lethal effects via the indirect exposure route of consuming contaminated prey.

Populations of parasitoid wasps often plummet due to emergence failure, as their larvae feed on hosts contaminated with insecticides or insect growth regulators. An example of the latter is pyriproxyfen, which does not affect adult insects but acts on their eggs and larvae. Thus, when presented with eggs of the brown stink bug, *Halyomorpha halys* (Hemiptera: Pentatomidae) produced by pyriproxyfen-treated females, the parasitoid wasp *Trissolcus japonicus* (Hymenoptera: Scelionidae) was observed ovipositing, but adult wasps failed to emerge from the host eggs [81]. Insect growth regulators can also reduce the survival of parasitoid wasps by altering the biochemistry and histology of the midgut epithelium, as is the case of lufenuron on the stink bug predator *Podisus nigrispinus* (Hemiptera: Pentatomidae), which is the main biological control agent of cotton leafworms, *Alabama argillacea* (Lepidoptera: Erebidae) [82].

The persistence of systemic insecticides in tissues of plants and fungi becomes a death trap for non-target mycophagous insects such as the twenty-spotted ladybeetle, *Psyllobora vigintimaculata* (Coleoptera: Coccinellidae), which feeds on conidia and hyphae of powdery mildews (Erysiphales). These pathogenic fungi grow on the treated plants and act as reservoirs of the insecticides applied (i.e., imidacloprid), indirectly poisoning the ladybugs that are the natural control of the fungi [83]. Similarly, systemic insecticides can adversely affect predatory bugs such as *Orius insidiosus* (Hemiptera: Anthocoridae), which often feed on plant sap [84], and the lacewing *Chrysoperla carnea* (Neuroptera: Chrysopidae), which also feeds on extra-floral nectar containing residues [85].

#### 2.2.4. Pathogens and Diseases in Pollinators

It has been suggested that the upsurge in the prevalence of pathogens and viral diseases among pollinators in recent decades is linked to the constant and increasing use of insecticides [86], and in particular neonicotinoids and fipronil [87,88]. Bees regularly ingest a cocktail of pesticides when feeding on pollen and nectar, as residues of these compounds are present not only in the treated crops but also in the flowers of weeds, shrubs and trees in the surrounding environment [89,90]. Although the doses ingested may be sublethal in most cases, they trigger detoxification mechanisms that are energy-draining and often result in stress [91]. This weakens the ability of the insects to fight against parasites, pathogens and diseases, as their immune system is compromised [92,93].

Not surprisingly, sublethal exposures of honey bees, *Apis mellifera* (Hymenoptera: Apidae) to the neonicotinoid imidacloprid in food significantly increased the infection by the microsporidian gut parasite *Nosema* spp. [94], which can be lethal if left unchecked [95]. Imidacloprid, however, did not affect the gut microbiome in bumblebees, *Bombus* sp. (Hymenoptera: Apidae), whereas several toxic metals did, indicating that such indirect effects are specific to the chemicals and species involved [96]. Honey bees that collected pollen and nectar from cornfields treated with thiamethoxam as seed-coatings had significantly higher pathogen loads of the mite *Varroa* sp. (Mesostigmata: Varroidae) than bees not exposed to the systemic insecticide. The mite weakened the bees considerably and reduced colony performance and survival [97]. Similarly, colonies of eastern bumblebees, *Bombus impatients* (Hymenoptera: Apidae) that pollinated blueberries treated with the neonicotinoid acetamiprid and the fungicide propiconazole had a higher infestation of the wax moth, *Vitula edmandsae* (Lepidoptera: Pyralidae) than colonies of the same bumblebees feeding on non-treated shrubs or treated only with one pesticide [98]. Areas of East Anglia treated with pesticides had low densities of insect pollinators and a high prevalence of Microsporidia parasites in leaf-cutter bees, *Megachile* sp. (Hymenoptera: Megachilidae), in contrast to the lower prevalence of three types of parasites among mason bees, *Osmia* sp. (Hymenoptera: Megachilidae), in the same region, which highlights again the different responses of individual species to the same insults [99].

### 2.3. Parasiticides

Livestock animals are often treated with parasiticides to protect them against external parasites such as lice, ticks and blowflies, as well as internal worms. It has been noticed for quite some time that residues of parasiticides in the dung of the treated animals may adversely affect pasture ecology [100], as the chemicals persist in the dung for days, weeks, or even months after treatment and, therefore, may directly and indirectly impact on the insects that depend exclusively on that particular micro-ecosystem. There are 450 species of coprophilous insects in North America and between 110 and 275 in Britain, and among them only five fly species that breed in undegraded dung pats are a nuisance to horses and cattle [101].

Among the various parasiticides used, avermectins and benzimidazole anthelmintics are persistent chemicals [102], and while avermectins are excreted mainly through faeces, benzimidazoles, organophosphates (OPs) and pyrethroids are excreted either in the urine or faeces [103]. Oral applications result in excretion within a week usually, but ivermectin residues can be released up to 5 months later [101]. 

Only a few indirect effects of parasiticides on insects associated with the dung microecosystem have been observed, as most reports focus on the direct effects only. For instance, after treating cattle with tetrachlorvinphos and diflubenzuron parasiticides, populations of the predaceous beetle *Sphaeridium scarabaeoides* (Coleoptera: Hydrophilidae) that feed on dung flies declined due to lack of food rather than to the direct toxicity of the parasiticides present in the dung [104]. Equally, parasitic wasps (Eucoilidae) and two species of *Cercyon* predaceous beetles (Coleoptera: Hydrophilidae) declined when their host fly larvae died from exposure to avermectin residues in dung [105]. Thus, livestock parasiticides applied over many years in the same areas eventually result in the decline of coprophilous insects by both direct and indirect effects on their populations [106]. After treating cattle with an annual dose of ivermectin in two successive years, the dung insect community declined in species abundance and richness [107]. However, long-term population impacts can be variable and depend mainly on (i) weather pattern during a given season, i.e., drought or wet; (ii) the proportion of treated livestock; and (iii) timing of application in relation to the oviposition life-cycle of the various species [101].

Another indirect effect of parasiticides is the slowdown of dung degradation. Since residues of these compounds have substantial adverse effects on the abundance or diversity of coprophilous insects, long-term changes in the processes of dung degradation can be expected. Apart from mortality, low residues produce a range of sublethal effects on the larvae of dung insects, with impacts on delayed development, reduced growth and emergence that inevitably results in lower degradation capacity [108,109].

As coprophilous insects differ markedly in their sensitivity to parasiticides, the indirect effects are better understood when the toxicity of the active ingredients towards the individual species is known. Briefly, pyrethroid ectocides are harmful to adults of the brown dung beetle, *Onthophagus gazella* (Coleoptera: Scarabaeidae) [110] as well as to larvae and adults of the common dung fly, *Neomyia cornicina* (Diptera: Muscidae) [109], while deltamethrin residues are harmful to larvae of the Australian bush fly, *Musca vetustissima* (Diptera: Muscidae) [111]. Flies are more susceptible to avermectins, OPs, diflubenzuron, methoprene and triflumuron than beetles, and Cyclorrhapha dipteran larvae are more susceptible than those of Nematocera due to earlier colonization of the dung pats, which results in higher residue doses being ingested. Coleoptera larvae are more susceptible than adults for similar reasons [101]. Avermectins are very toxic to flies and beetles alike even several months after treatment [112,113]; residues of ivermectin from sustained-release devices can kill parasitoid wasp larvae and coprophagous beetles up to 128 days post-treatment [114]. However, moxidectin is not toxic to coprophilous insects [115], nor are the benzimidazoles and other anti-helminthic parasiticides, except oxfendazole [103]. For a comprehensive review on the direct effects of parasiticides on coprophilous insects see Floate et al. [101].

### 2.4. Fungicides

Fungicides can be highly toxic to a wide range of organisms. This is because many fungicides are broad-spectrum biocides as their mode of action often involves inhibition of cellular respiration or division which are common to all organisms. Many fungicides are applied prophylactically, either as seed coatings or sprays on the crops. A large proportion is applied to fruits, particularly vineyards, using vertical sprays, and this increases their risk of drifting into adjacent land and surface waters. Studies on the effects of fungicides on arthropod communities are lacking. However, laboratory experiments with 4 fungicides (i.e., aluminium tris, azoxystrobin, fenhexamid and kresoxim-methyl) showed they had no indirect effects on the predatory flower bug, *Orius insidiosus* (Hemiptera: Anthocoridae), whereas 8 insecticides significantly reduced the survival of the insects [116].

Application of fungicide affects the density of fungivorous mites but might also have an indirect effect on predatory mites (Acari: Phytoseiidae) by reducing their food availability, as some are known to feed on fungal pathogens like mildew [117]. Moreover, fungicides reduce entomopathogenic fungi, which naturally occur in soil and phyloplan and are natural agents suppressing many arthropod pests. Application of fungicides can thus have a positive indirect effect on herbivores.

### 2.5. Pesticide Mixtures

Populations of natural enemies of pest crops such as parasitoids, predatory mites, hunting spiders, ladybugs, rove beetles and carabids, are typically reduced by pesticide applications and indirectly help increase herbivorous pest species. Seed-treatments with neonicotinoid insecticides (i.e., thiamethoxan, imidacloprid) and fungicides (i.e., fludioxonil, mefenoxam, sedaxane, thiabendazole and azoxystrobin) directly eliminate larvae of insects which feed on seeds of crop weeds, thus increasing the weed soil bank and reducing their diversity significantly [118].

A meta-analysis of 685 arable fields treated with different combinations of pesticides confirmed that restriction of pesticide inputs benefits arthropod populations at the edges of arable fields, but the main changes are due to herbicides. Increases of Staphylinidae, Neuroptera and some Diptera groups were due only to restriction of herbicides. Restricted use of herbicides, insecticides and fungicides resulted in increased abundance of Heteroptera up to 13 times and four Coleoptera orders up to 9 times higher than in treated fields. For other invertebrates, restricted use of pesticides generally either increased the abundance of arthropods (i.e., Lepidoptera and Symphyta) or had little or no significant impact (i.e., Carabidae and spiders) [119].

## 3. Aquatic Ecosystems

Arthropods in aquatic ecosystems include a variety of insects in different trophic levels: grazers like chironomids (Diptera); scrapers and detritivorous larvae of mayflies (Ephemeroptera), stoneflies (Plecoptera) and caddisflies (Trichoptera); scavenger beetles (Coleoptera: Hydrophilidae); and predators such as damselflies and dragonflies (Odonata), alderflies and dobsonflies (Megaloptera), crane flies (Diptera: Tipulidae), backswimmers (Hemiptera: Notonectidae), water scorpions (Hemiptera: Nepidae), water striders (Hemiptera: Gerridae) and diving beetles (Coleoptera: Dytiscidae). 

**Figure 2:**
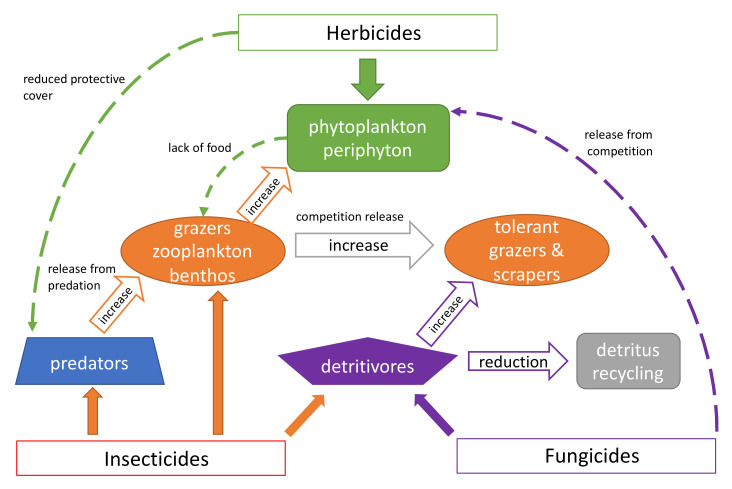
Indirect effects of pesticides on aquatic ecosystems are indicated by hollow and dashed arrows and direct effects by solid arrows.

While the direct effects of pesticides on the above taxa are well known [120,121,122,123], there is much less information about the indirect effects these chemicals have on the diversity of aquatic insects. A former review on indirect effects dealt mainly with other aquatic organisms [13]. Most of what we know refers to other aquatic arthropod taxa such as detritivorous amphipods and isopods (Crustacea) and small grazing zooplankton and epibenthic species (Crustacea: Cladocera, Copepoda and Ostracoda). As indirect effects are the consequence of trophic alterations regardless of taxonomic groups, and bearing in mind that the sensitivity of arthropods towards pesticides is very similar, it is appropriate to consider the indirect impacts of pesticides on all arthropods together (Figure 2).

### 3.1. Insecticides

Insecticides are very toxic to all aquatic arthropods, particularly to zooplankton and insect larvae [124,125]. Residues of insecticides in streams are at higher levels and more common near agricultural fields than in rivers and lakes, as these chemicals dissipate rather quickly from water. The exception are systemic insecticides, which are water-soluble and persistent in all these media [126].

Impacts of insecticides on aquatic communities are usually due to direct effects on zooplankton crustaceans [127,128,129] and insect larvae [130,131], all of which are very susceptible to neurotoxic chemicals. However, the reduction in grazer arthropods indirectly boost the growth of producers such as algae and periphyton [132,133,134], and also allows tolerant herbivore species of copepods, worms and molluscs to thrive [135,136]. Moreover, direct reductions in predatory insects such as water bugs and dragonfly nymphs typically lead to increases in the abundance of their prey, i.e., tadpoles and snails that benefit from this indirect effect [11]. Indeed, dragonfly nymphs pose a significant threat to amphibian larvae in aquatic communities and are capable of reducing tadpole biomass by >80% in a two-week period, so when the dragonflies were eliminated by the insecticide endosulfan in controlled mesocosms the tadpoles increased in numbers [137].

Mixtures of insecticides can have devastating effects on susceptible benthic arthropods and zooplankton species. For example, a mixture of permethrin, λ-cyhalothrin and chlorpyrifos reduced dramatically the density of the waterflea *Daphnia magna* (Crustacea: Cladocera) and the amphipod *Hyalella azteca* (Crustacea: Amphipoda) within 24 h after application [135]. The lethal effect of four insecticides (chlorpyrifos, diazinon, endosulfan and malathion) applied at 10 or 40 μg/L on waterfleas and copepods induced trophic cascades that facilitated algal blooms and abiotic changes. While the effect of the OPs was swift, endosulfan produced a lag effect by reducing the abundance of waterfleas and amphibians. The mixture treatment had lethal effects throughout the community that led to long-term effects on amphibian mass and unique indirect consequences on phytoplankton and water quality variables such as pH, dissolved oxygen and turbidity [138].

The magnitude of the impacts depends largely on the properties of the individual chemicals [139]. For example, malathion and carbaryl have the same effects on aquatic communities, but effects of the former are more persistent than those of the latter because the toxicity of OP insecticides last longer than that of carbamates [140]. Concentrations of chlorpyrifos as low as 1 μg/L can significantly reduce the zooplankton communities, although the impacts are lower under higher temperatures due to the dissipation of the insecticide [141]. Structural characteristics of the aquatic bodies can also modify the overall impacts of insecticides. For example, using a factorial design in mesocosms, Brogan et al. [142] showed that macrophyte density protected zooplankton and other animal taxa against malathion spraying effects. Thus, species richness and abundance of spiders along Romanian streams were negatively associated not only with in-stream pesticide toxicity but also with the shading of the stream bank due to vegetation cover, a proxy for the quality of the habitat [143].

Combined direct and indirect effects often result in long-term changes at the community and ecosystem level of organisation even after a single pulse of non-persistent insecticides [136], but more severe indirect effects and longer recovery periods of the affected populations occur under repeated applications [144,145]. Such effects reduce energy transfer efficiency, elongate the food chain and sometimes increase species richness [127]. Eventually, the continuous disturbance of the aquatic food webs can ultimately lead to the collapse of entire fisheries that depend on invertebrate food sources, as it occurred in Lake Shinji (Japan) when imidacloprid residues that drained into the lake over the years eliminated the insects and zooplankton [146].

It appears that release from competition among species with different sensitivity is the major indirect effect of insecticides. For example, the elimination of sensitive grazing waterfleas (Cladocera) often results in increases in copepods and Rotifera species due to a release from competition with waterfleas [127,128,129]. Similarly, the elimination of various predatory arthropods after treatment with fenvalerate allowed the worm *Stylaria lacustris* (Oligochaeta) to increase in abundance [136]. Dinotefuran applied to experimental rice mesocosms at the recommended rates (10 kg/ha) increased the abundance of some insects, particularly chironomid larvae and nymphs of the dragonfly *Crocothemis servilia* (Odonata: Libellulidae) due to a combined indirect effect from the lack of competition with other dragonfly species and increased chironomid prey [147]. In this context, it is important to know the individual species sensitivities for predicting the possible indirect effects of insecticidal compounds. Some indicator taxa are water striders, *Gerris lacustris* (Hemiptera: Gerridae), dragonfly nymphs of *Orthetrum albistylum* (Odonata: Libellulidae) and diving beetles such as *Hydroglyphus japonicus* (Coleoptera: Dytiscidae) for exposures to clothianidin, fipronil or chlorantraniliprole, respectively [148].

Compensatory effects occur due to the differential toxicity and tolerance of individual species in the ecosystem. For example, the decrease in numbers of *Streblocerus pygmaeus* (Crustacea: Cladocera) by chlorpyrifos was compensated by increases in a more tolerant congener such as *Dunhevedia crassa* (Crustacea: Cladocera) [145]. The influence of intraspecific competition on a detritivorous caddisfly, *Limnephilus lunatus* (Trichoptera: Limnephilidae) was demonstrated in mesocosms treated with the pyrethroid fenvalerate. The compensation of direct effects by the chemical at high concentrations was due to a reduction of intraspecific pressure in the population, whereas at low concentrations (≤0.1 μg/L) the effects of the toxicant were not compensated [149]. Fenvalerate also caused high mortality in populations of *D. magna* in a microcosm, but the waterfleas recovered quickly in both numbers and biomass within 2 weeks; nevertheless, the recovery was faster and achieved higher biomass under low intraspecific competition than under high competition [150].

Leaves and other vegetable matter that fall into streams and rivers are consumed by a large array of detritivorous arthropods, including larvae of mayflies, stoneflies and caddisflies as well as amphipods. Senescent leaves of trees that had been treated with the systemic insecticide imidacloprid to control the Asian longhorn beetle, *Anoplophora glabripennis* (Coleoptera: Cerambycidae), contained residue levels sufficient to reduce significantly the natural decomposition processes carried out by the stonefly *Pteronarcys dorsata* (Plecoptera: Pteronarcyidae) and nymphs and the crane fly *Tipula* sp. (Diptera: Tipulidae) in aquatic microcosms, even if the insects did not die [151]. However, higher concentrations of imidacloprid in water (15 μg/L) led to the starvation of the amphipod *Gammarus pulex* (Crustacea: Amphipoda) by directly inhibiting its feeding on the litter [152]. In microcosms treated with thiacloprid, *G. pulex* was able to feed on leaf litter and prey on nymphs of the mayfly *Baetis rhodani* (Ephemeroptera: Baetidae) up to 1 μg/L; however, at higher concentrations (4 μg/L) the amphipod reduced its leaf consumption and stopped predation on the mayflies [153]. Thus, sublethal impacts of such insecticides on detritivorous arthropods can result in impairment of trophic relationships and reductions in the decomposition and nutrient recycling processes in aquatic systems [154].

Another indirect effect is the enhancement of insecticidal effects by predation. For instance, the insecticide chlorpyrifos at 1 μg/L directly reduced the biomass of herbivorous plankton (4 waterflea species) by 7–12% in microcosms, with the smallest decrease when all species were present. The introduction of a predatory glassworm, *Chaoborus obscuripes* (Diptera: Chaoboridae) increased the total impact of the insecticide on the waterfleas [18]. Apart from the direct effects of imidacloprid on benthic macroinvertebrate assemblages [155], exposure of the caddisfly *Sericostoma vittatum* (Trichoptera: Sericostomatidae) and the midge *Chironomus riparius* (Diptera: Chironomidae) to sublethal levels of this insecticide also compromised antipredator behavioural responses in both insect species [156]. While chlorantraniliprole reduced the decomposition of leaves carried out by the shredder caddisfly *S. vittatum*, as well as the growth of midge *C. riparius*, these effects were enhanced in the presence of predation by nymphs of the golden-ring dragonfly, *Cordulegaster boltonii* (Odonata: Cordulegastridae) [157]. The combined direct and indirect effects of sublethal concentrations of pesticides can have negative consequences in terms of mortality from predation in benthic insect populations, and also induce maladaptive responses among zooplankton species which may reduce their long-term viability in the field [158].

### 3.2. Fungicides

Fungicides are very toxic to arthropods and other aquatic organisms. In European surface waters, fungicides dominate the residue levels (median 0.96 μg/L) compared to herbicides (median 0.063 μg/L) and insecticides (median 0.034 μg/L). A comprehensive review of the risks that such contamination poses to aquatic ecosystems can be found in Zubrod et al. [159].

A commonly reported indirect effect of fungicides is an increase in phytoplankton abundance or biofilm biomass. This could be due to (a) reduced grazing pressure by affected invertebrate consumers, mainly zooplankton species and chironomids or (b) microorganisms benefiting from the metabolization of organic material set free by dying organisms. In some studies, interactions within microbial communities result in increasing diatoms and decreasing protozoans [160].

For example, mesocosms treated with pentachlorophenol at 121 μg/L increased the abundance of the alga *Cryptomonas* sp. (Cryptophyceae) due to reduced grazing pressure, reduced competition, or increased decomposition of the fungicide. These effects were evident also at lower treatment levels in autumn but not in winter [161]. In Thailand, mesocosms treated with carbendazim produced blooms of the floating macrophyte *Wolffia* sp. (Alismatales: Araceaae) due to the reduction or elimination of zooplankton rotifers (*Keratella tropica*), waterfleas (*Moina micrura, Ceriodaphnia cornuta* and *Diaphanosoma* sp.) and cyclopoid copepods. These changes resulted also in altered water conditions, which became anoxic during the last three weeks of the experiment [162]. The dithiocarbamate fungicide metiram applied to microcosms up to 324 μg/L also produced increases in phytoplankton due to the reduction in densities of grazing rotifers and copepods, as these were the most sensitive taxa towards the fungicide [163].

Another common indirect effect is the increase in abundance of tolerant macroinvertebrates, which results from either reduced predation pressure or release of competition with species in the same trophic guild that are more susceptible to the fungicide, or both. In microcosms treated with the dithiocarbamate fungicide metiram at various concentrations (4 to 324 μg/L), population densities of a few macroinvertebrates increased in the short-term, although they were not consistent with the concentrations used. This unexpected outcome was likely due to shifts in species interactions as a result of direct toxic effects of the fungicide on susceptible species such as predatory beetles (Dytiscidae) and *Caenis* sp. mayflies (Ephemeroptera: Caenidae) [163]. Similarly, a concentration of carbendazim at 1000 µg/L applied in microcosms resulted in increases of the snail *Lymnaea* sp., as the flatworm predator *Girardia tigrina* was reduced in numbers together with macroinvertebrate herbivores that exploited the same niche (i.e., oligochaetes, the crustaceans *Gammarus juvenile* and *G. pulex* and the molluscs *Bithynia tentaculata* and *B. leachi*) [164]. The same indirect effect was observed in tropical mesocosm treated with this fungicide [162].

A third indirect effect of fungicides on aquatic ecosystems is the alteration of saprophytic function in the aquatic ecosystem, carried out mainly by detritivorous crustaceans and larvae of mayflies, caddisflies and stoneflies, as well as by bacteria consortia [165,166]. As with insecticides, this effect is the consequence of the direct reduction of populations of these benthic invertebrates due to the toxicity of the fungicides. Measuring this functional disturbance can only be done in mesocosms and microcosms. For example, the fungicide thiram at 35 and 170 μg/L applied to stream mesocosms and ponds reduced the overall litter break-down a few weeks after the treatment, as the detritivorous asellids (Isopoda) and gammarids (Amphipoda) were eliminated in large numbers over that period [167]. Similarly, the reduced decomposition of banana leaves observed eight weeks after application of carbendazim in tropical mesocosms was considered to be the indirect effect of a decreased microbial activity that resulted from the anoxic water conditions created by algae blooms [162]. This effect may be also due to avoidance of the litter that contains fungicide residues, as several other mesocosm studies indicate. In choice experiments with leaves of black alder (*Alnus glutinosa*) treated or not with tebuconazole at 50 and 500 μg/L, *Gammarus pulex* (Crustacea: Amphipoda) significantly preferred untreated leaves over those treated with the fungicide. It appears that the fungicide eliminated fungal species such as *Alatospora acumunata, Clavariopsis aquatica* or *Flagellospora curvula,* which are preferred by the amphipod [168]. Indeed, the nutritional quality of the litter leaves matters to the shredders to the extent that in another experiment, that amphipod and the caddisfly *Halesus radiatus* (Trichoptera: Limnephilidae) consumed significantly more leaves that had lower microbial biomass as a result of having been treated with the fungicide propiconazole, in order to compensate for the reduced nutritional quality of the litter [169]. In another experiment, *G. pulex* showed a preference for black alder leaves that were treated with a mixture of fungicides (azoxystrobin, cyprodinil, quinoxyfen and tebuconazole at recommended field rates), probably because this treatment reduced pathogenic fungi while allowing the growth of other microbial and fungal communities that were more palatable to the amphipods. This shift in fungal community composition and increased nutritional quality and palatability of leaf material ultimately resulted in a higher gammarid growth (up to 300% increase) during a 24-day long-term feeding assay [170].

### 3.3. Herbicides

Herbicides are the most common pesticides found in freshwaters [171]. While they are not as toxic to animals as insecticides and fungicides are, the presence of a large cocktail of plant killers in aquatic ecosystems cannot be overlooked, as algae and communities of aquatic macrophytes can be seriously affected.

Two main indirect effects of herbicides have been observed in experimental ecosystems. The first is a decrease in the abundance of grazing invertebrates as a consequence of a reduction in phytoplankton and/or periphyton by the direct herbicidal toxicity. For example, the herbicide terbutryn applied to microcosms up to 6 μg/L eliminated the periphyton food source of the grazer mayfly *Rhithrogena semicolorate* (Ephemeroptera: Heptageniidae), which larvae decreased significantly in numbers when compared to untreated controls [172]. Atrazine in experimental ponds at various concentrations decreased the abundance of chironomids and other herbivorous insects presumably through reduction of the periphyton food source and, to some extent, their habitat. The abundance of detritivorous insects such as the caddisfly *Oxyethira pallida* (Trichoptera: Hydroptilidae) was not affected, although early emergence was observed, and populations of predatory insects were not affected. However, the species richness and evenness of the pond was reduced [173]. Microcosms treated with the herbicide linuron at 100 μg/L reduced the biomass of five species of algae by 17%, and a further reduction of 42% was observed when four species of herbivorous zooplankton were introduced into the system. The highest effects occurred after 6 days and then declined as the herbicide dissipated and the algae recovered [18]. Interestingly, the total impact of the herbicide in the system was mitigated with the introduction of the predatory glassworm *Chaoborus obscuripes* (Diptera: Chaoboridae), which alleviated the grazing pressure on the phytoplankton and allowed it a fast recovery. This effect has also been observed in field situations. Typical combinations of herbicides applied routinely to rice fields in Japan have been reported as reducing the abundance of grazing worms (Oligochaeta) and other herbivorous invertebrate taxa, as their periphyton food source is almost eliminated by the direct action of the herbicides. The only taxon that does not appear to be affected by the herbicides is midges (Chironomidae), perhaps because they can also feed on periphyton. However, as in the microcosm experiments, further reductions in both worms and chironomids occur when predatory insects are present in the fields [174].

Another indirect effect is the reduction of available refugia for predatory insects, as herbicides can reduce significantly the biomass of macrophytes [173]. This effect is more subtle and not as evident as the former but has been demonstrated in mesocosms with macrophytes treated with the herbicide pentoxazone. The herbicide did not affect the phytoplankton and consequently, there were no clear negative impacts on zooplankton nor on herbivorous and detritivores insects. However, as the herbicide significantly reduced the biomass and surface cover of the aquatic plants, nymphs of the dragonfly *Orthetrum albistylum* (Odonata: Libellulidae) were notoriously absent from the treated plots. Lack of protection by the aquatic plants resulted also in smaller decreases in abundance in other insect predators [175]. In contrast, mesocosms with dense stands of aquatic plants reduced the abundance of periphyton and hence lowered the numbers of grazing snails and tadpoles [142]. Differences in macrophyte density and associated invertebrate communities between channels treated or not with herbicides had already been reported in agricultural settings [176].

### 3.4. Pesticide Mixtures

Mixtures of pesticides can have compensatory effects or else result in additive negative impacts. Ecological theory can help predict the direction of effects of multiple chemical mixtures by integrating information on each functional group’s (1) sensitivity to the chemicals (direct effects), (2) reproductive and recovery rates, (3) interaction strength with other functional groups (indirect effects) and (4) links to ecosystem properties [177]. The initial composition of the community usually influences the direction of the combined pesticide effects.

An example of additive negative effects is when the herbicide atrazine is applied together with the insecticide terbufos in a microcosm. The herbicide at 15 μg/L reduced the algae density significantly and indirectly led to a reduction in waterfleas and chironomids abundance, whereas terbufos (at 0.1 or 10 μg/L) directly suppressed the latter two taxa due to its high toxicity [178]. Equally, in mesocosms treated with atrazine (25 μg/L) and endosulfan (10 μg/L) in two pulses, atrazine directly decreased periphyton and this effect indirectly reduced chironomid abundance, while endosulfan reduced chironomid larvae dramatically, which resulted in indirect increases of snails and decreases in competing tadpoles [12]. Effects may not be always noticeable when the pesticides in water are present at sublethal concentrations to both the algae and the grazers, as it often occurs with the herbicide diuron and the insecticide imidacloprid. The combination of these two pesticides at relevant environmental concentrations (5 μg/L) in microcosms, however, altered the nutritional behaviour of chironomid larvae, with imidacloprid leading to an inhibition of grazing activity, while diuron provoked a nutritional quality loss of algae which probably affected their palatability [179].

Finally, a comprehensive mesocosm study on the impacts of 2 insecticides (carbaryl and malathion) mixed with 2 herbicides (glyphosate and 2,4-D) on non-target organisms was carried out after application at the recommended commercial rates. Among the 25 species in the mesocosms were six predatory insects: water bugs *Notonecta* and *Belostoma* (Hemiptera) and nymphs of *Anax* and *Tramea* dragonflies (Odonata) that prey on both tadpoles and snails, and larval predatory beetles (Coleoptera: Dytiscidae) such as *Dytiscus* sp. that prey on tadpoles and *Acilius* sp. that prey on zooplankton. As it could be expected, the insecticides reduced the diversity and biomass of zooplankton and predatory insects, which indirectly increased the abundance of several species of tadpoles due to a lack of predation. The two herbicides, however, did not reduce the periphyton biomass and had no effects on zooplankton, insect predators, nor snails [129].

## 4. Conclusions

Pesticides have a wider range of effects on arthropods than expected by considering only their toxicity to their target pests or weeds. In terrestrial ecosystems, both insecticides and herbicides help increase indirectly the populations of herbivorous insects, many of which are pests or become pests as a result of their applications. In aquatic ecosystems, insecticides and fungicides can indirectly cause algae blooms or increase the biomass of periphyton as the grazers are reduced in numbers or eliminated. At the same time, the abundance of herbivore species of arthropods and other animals (e.g., snails and tadpoles) that are tolerant to the chemicals may increase due to the combined effect of a reduction in predators and an increase in producers biomass. Herbicides reduce the biomass and surface cover of aquatic plants, which may indirectly affect populations of insect predators and other animals.

In all cases, the changes in arthropod populations are the result of ecological interactions among the species affected. The main mechanism appears to be the release of herbivores from predation or parasitism, although the release from competition with other species in the same niche also plays a crucial role. An understanding of the indirect effects of pesticides is important for effective control of pests, weeds and diseases, as all too often the application of chemicals produces the opposite effects they were intended to have. Integrated pest and weed management programs should learn from these lessons so as not to repeat the mistakes of the past.

## Data Availability

Not applicable.
